# Diagnostic Dilemma in Groove Pancreatitis: A Case Report

**DOI:** 10.31729/jnma.5301

**Published:** 2020-11-30

**Authors:** Uttam Laudari, Roshan Ghimire, Rosi Pradhan, Dhiresh Kumar Maharjan, Prabin Bikram Thapa

**Affiliations:** 1 Department of Surgery, Hospital for Advanced Medicine and Surgery, Dhumbarahi, Kathmandu, Nepal; 2 Department of Surgery, Kathmandu Medical College, Sinamangal, Kathmandu, Nepal; 3 Department of Anaesthesiology, KIST Medical College, Imadol, Lalitpur, Nepal

**Keywords:** *chronic pancreatitis*, *pancreaticoduodenectomy*, *pancreatitis*

## Abstract

Groove pancreatitis is an uncommon form of chronic pancreatitis common in patients with a history of smoking and alcohol abuse. A high index of suspicion is required as it may masquerade pancreatic ductal adenocarcinoma and both of these conditions are difficult to differentiate preoperatively. Pancreaticoduodenectomy has a good outcome in a patient with Groove pancreatitis. Hence, we are reporting a case report of Groove pancreatitis in 40 years gentleman, who was being managed by multiple endoscopic dilatations, later underwent pancreaticoduodenectomy for persistent symptoms. He had no perioperative morbidity and doing well in 24 months follow up.

## INTRODUCTION

Groove Pancreatitis (GP) was first described by Becker in 1973 that affects the anatomical area between the pancreatic head, the duodenum, and the common bile duct.^1^ GP is commonly seen among middle-aged men who abuse alcohol. Patients present with weight loss, upper abdominal pain associated with nausea, and postprandial vomiting. Radiological investigation cannot always distinguish GP with pancreatic cancer so a high index of suspicion is required. Most patients ultimately undergo pancreaticoduodenectomy with a favorable outcome.^2,3^ Our case highlights the diagnostic challenges and a favorable outcome of pancreaticoduodenectomy in a patient with GP.

## CASE REPORT

**Figure 1:**
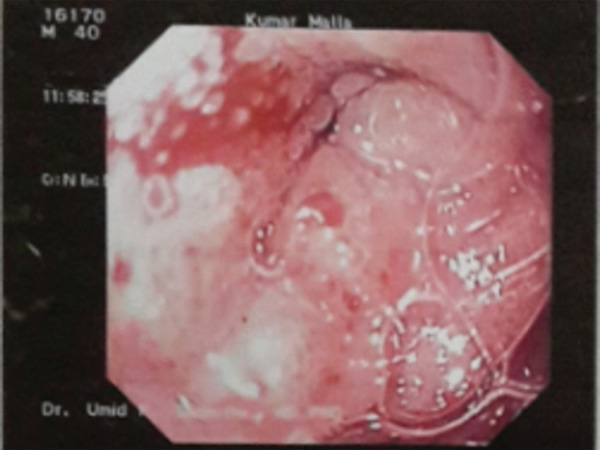
Stenosis of the first part of Duodenum.

A forty-year-old gentleman presented to the outpatient department with gradually progressive postprandial nausea and vomiting associated with upper abdominal pain for three months and significant weight loss of >10kg in the last six months. Upper gastrointestinal endoscopy revealed stenosis of the first and second part of the duodenum (Figure 1).

**Figure 2:**
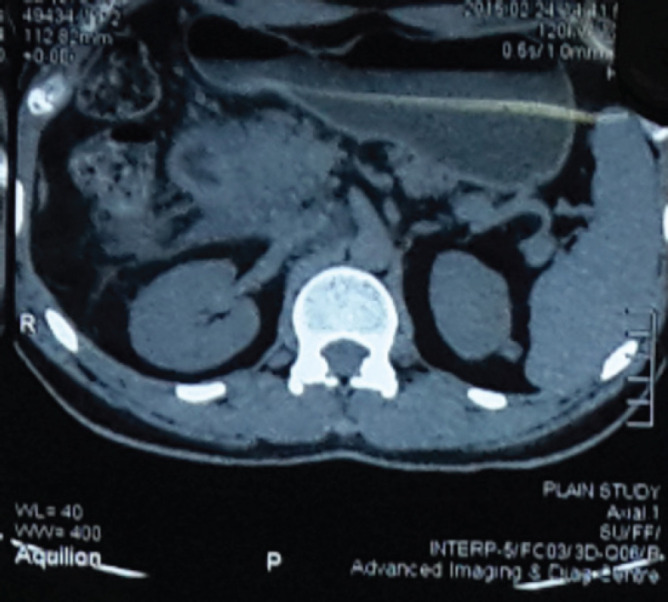
CECT abdomen Enhancing irregular circumferential wall thickening of D1 D2 junction with luminal narrowing.

He had undergone three episodes of endoscopic duodenal dilatation with minimal benefit. Contrast-enhanced computed tomography scan (CECT) showed enhancing irregular circumferential wall thickening of at the junction of the first and second part of the duodenum with luminal narrowing, swollen pancreatic head, and uncinate process suggesting either benign or malignant duodenal stricture with acute pancreatitis (Figure 2).

**Figure 3:**
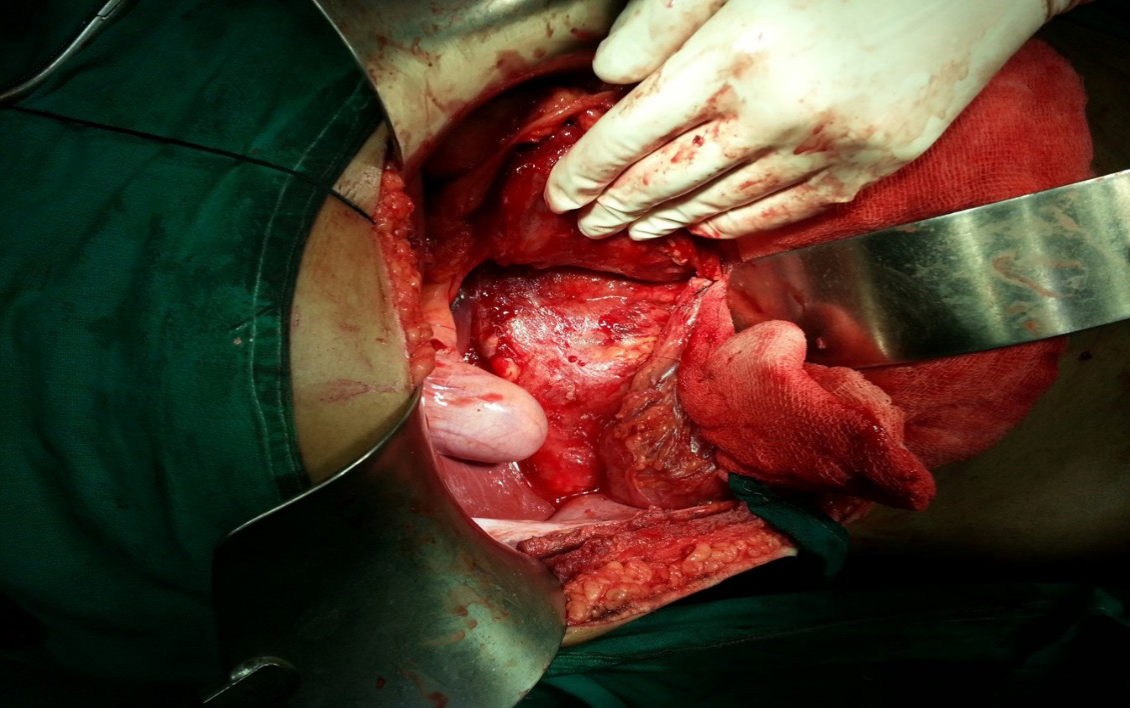
Enlarged hard pancreatic head and thickened duodenal wall.

Laboratory investigations were within normal limits and CA-19.9 value of 92.5U/ml. He had a normal IgG4 level. After a series of interventions and medical management associated with persistent severe symptoms, he underwent a pancreaticoduodenectomy (Whipple procedure). Intraoperatively, there was no evidence of local invasion, any organ invasion, metastatic deposits with enlarged hard pancreatic head, and thickened duodenal wall (Figure 3).

**Figure 4:**
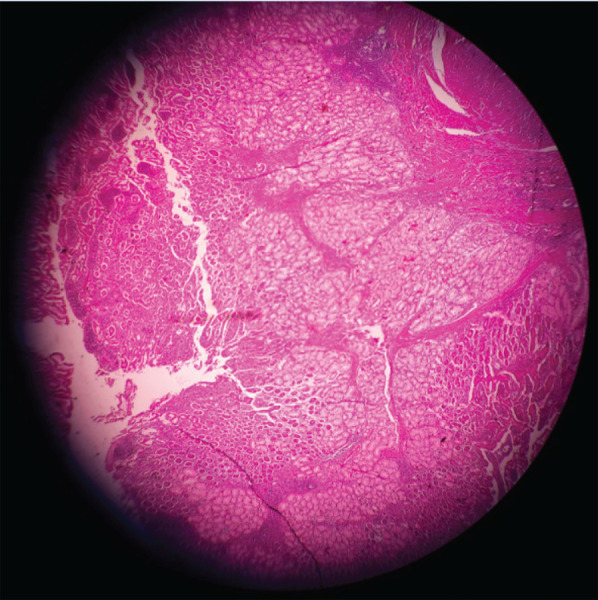
Distorted duodenum, fibroses, and hypertrophied duodenal mucosa, hyperplastic Brunner's gland.

Histopathology revealed distorted duodenum, fibroses, and hypertrophied duodenal mucosa, hyperplastic Brunner's gland (Figure 4).

Clinical, biochemical, radiological, and pathological findings were consistent with Groove Pancreatitis. Postoperative recovery was uneventful. The patient is doing well at 24 months follow-up.

## DISCUSSION

Out of three patients who underwent Whipple pancreaticoduodenectomy for GP, the present case is our first case who had repeated hospital admission for frequent vomiting and upper abdominal symptoms successfully managed with surgery and doing fine in 24 months after follow up.

The pancreaticoduodenal groove is a space formed medially by the pancreatic head, laterally by the second portion of the duodenum, posteriorly by the third part of the duodenum and inferior vena cava and duodenal bulb on the superior aspect. The space has distal CBD, main pancreatic duct (MPD), accessory pancreatic duct, major papilla and minor papilla with associated small arteries (superior pancreaticoduodenal artery) and lymph nodes. GP is one of the differentials which our patient was managed successfully with Whipple's pancreaticoduodenectomy.^3^

Arora et al., found all 33 patients in their study were male with a mean age of 46.27 years. Among 33 patients, 31 patients were long term alcohol consumption and 14 patients had a history of smoking.^4^ Aguilera et al also presented a total of eight cases where the male to female ratio was an equal and predominant history of alcohol and smoking.^5^ Both alcohol and tobacco increase viscosity of pancreatic juice and pancreatic duct obstruction. In long-term leading to fibrosis and calcification of duct of Santorini and minor papilla, surrounding the pancreatic head and duodenal wall leading to scarring and stenosis of the duodenum.^1^ Patient with GP present with gradual worsening or acute episode of abdominal pain associated with nausea and vomiting, Other less common presenting complaints were weight loss, features of gastric outlet obstruction and obstructive jaundice, underlying alcohol-related liver cirrhosis.^4^ Serum amylase and lipase may be mildly elevated or within the normal range. Marker of abdominal malignancy CEA and CA-19-9 are usually normal or mild elevation. Very rarely patients may present with the feature of pancreatic insufficiency as large part of the gland is spared by disease. Our patient was middle-aged with a similar history of alcohol and smoking who had upper abdominal symptoms, weight loss, normal enzymes level, normal tumour markers and no any feature of pancreatic insufficiency being managed repeatedly with endoscopic dilatation for duodenal stricture.

Pancreatic protocol CECT abdomen may show two variants of GP either “cystic” where multiple Para duodenal or duodenal cysts protrudes into the duodenal wall which is a valuable indication for diagnosis or “Solid” type where “sheet-like” curvilinear crescentic solid mass with medial duodenal wall thickening is seen. This sheet of solid mass looks hypoattenuating after contrast enhancement and on delayed imaging, due to its fibrous nature may show mild enhancement. In the pure form of GP sheet-like fibrotic scarring is confined within the groove whereas in the segmental form it extends to involve the head of the pancreas, MPD causing stenosis, sometimes forming mass-like lesion which may mimic scirrhous pancreatic ductal adenocarcinoma (PDAC) and very difficult to differentiate radiologically. Our case was a segmental form involving head and MPD. Unlike PDAC in GP peripancreatic vessels are neither constricted, attenuated nor encased. Identification of normal-sized gastroduodenal artery (GDA) which is displaced leftward favors a diagnosis of GP whereas in PDAC it is maybe encased or constricted by soft tissue.^3,4^ Associated scarring, luminal compromise, and upstream gastric dilatation may be seen. Magnetic resonance imaging (MRI) of abdomen findings mirror that those of CECT finding. CECT abdomen of our case couldn't differentiate PDAC with GP. The involvement of the pancreas in segmental GP is more clearly visible in the MRI. Magnetic resonance cholangiopancreatography (MRCP) better delineates distal CBD and pancreatic duct which may be narrowed, also the distance between the ampulla and the duodenal lumen is widened in GP. Distal CBD narrowing and stricture of ampulla may show dilated “Banana Shaped” gall bladder.^3^ Endoscopic ultrasound (EUS) delineates all the layers of the intestine and much information regarding the duodenum and adjacent structures. It gives supplementary information other than CECT and MRI and also aids in guided biopsy to rule out malignancy. Abrupt irregular narrowing of MPD with significant upstream dilatation is diagnostic of PDAC whereas smooth progressive narrowing is usually benign conditions.^4^

Management of GP is can be done conservatively, endoscopically, and with surgical resection.^2^ Acute phase is treated with analgesics and nutrition and abstinence from alcohol and smoking.^6^ Endoscopic stenting of minor papilla also has been done but a long-term outcome is yet to be established.^6,7^ Most patients who fail medical therapy are managed with Pancreaticoduodenectomy due to marked duodenal stenosis, endocrine and exocrine failure, progressive pain, and when malignancy cannot be ruled out.^8^ Aguilera et al treated eight patients with pylorus-preserving pancreaticoduodenectomy by both open and laparoscopic approach with no major complications and they found the resolution of pancreatitis and improvements in symptoms in during median follow up 18.15 month.^5^ Out of three cases we operated for GP, the present case is our first case who is doing fine with no any symptoms during 24 months of follow up, results of long term follow up of remaining two cases are yet to be confirmed.

Groove pancreatitis is an uncommon variant of chronic pancreatitis. Differentiating this condition with other common causes of chronic pancreatitis and pancreatic cancer is challenging based on clinical examination and radiological findings. Medical and endoscopic management have been described but with the availability of surgical expertise, pancreaticoduodenectomy is a safe modality treatment with acceptable outcomes.
